# Umbilical Cord Blood Leptin and IL-6 in the Presence of Maternal Diabetes or Chorioamnionitis

**DOI:** 10.3389/fendo.2022.836541

**Published:** 2022-02-07

**Authors:** Lauren K. Vasilakos, Baiba Steinbrekera, Donna A. Santillan, Mark K. Santillan, Debra S. Brandt, Daniel Dagle, Robert D. Roghair

**Affiliations:** ^1^ Stead Family Department of Pediatrics, Carver College of Medicine, University of Iowa, Iowa City, IA, United States; ^2^ Department of Pediatrics, University of South Dakota, Sioux Falls, SD, United States; ^3^ Department of Obstetrics and Gynecology, Carver College of Medicine, University of Iowa, Iowa City, IA, United States

**Keywords:** development, inflammation, insulin, neonatal, obesity, sepsis, adipokine cytokine

## Abstract

Diabetes during pregnancy is associated with elevated maternal insulin, leptin and IL-6. Within the placenta, IL-6 can further stimulate leptin production. Despite structural similarities and shared roles in inflammation, leptin and IL-6 have contrasting effects on neurodevelopment, and the relative importance of maternal diabetes or chorioamnionitis on fetal hormone exposure has not been defined. We hypothesized that there would be a positive correlation between IL-6 and leptin with progressively increased levels in pregnancies complicated by maternal diabetes and chorioamnionitis. To test this hypothesis, cord blood samples were obtained from 104 term infants, including 47 exposed to maternal diabetes. Leptin, insulin, and IL-6 were quantified by multiplex assay. Factors independently associated with hormone levels were identified by univariate and multivariate linear regression. Unlike IL-6, leptin and insulin were significantly increased by maternal diabetes. Maternal BMI and birth weight were independent predictors of leptin and insulin with birth weight the strongest predictor of leptin. Clinically diagnosed chorioamnionitis and neonatal sepsis were associated with increased IL-6 but not leptin. Among appropriate for gestational age infants without sepsis, IL-6 and leptin were strongly correlated (R=0.6, P<0.001). In summary, maternal diabetes and birth weight are associated with leptin while chorioamnionitis is associated with IL-6. The constraint of the positive association between leptin and IL-6 to infants without sepsis suggests that the term infant and placenta may have a limited capacity to increase cord blood levels of the neuroprotective hormone leptin in the presence of increased cord blood levels of the potential neurotoxin IL-6.

## Introduction

Cord blood analysis is used to identify potential links from intrauterine exposures to long-term outcomes. The cord blood levels of some hormones, such as insulin, primarily reflect production by the fetus (1), while other hormones, including the adipokines leptin and IL-6 (2), can enter the fetal circulation from the placenta (3, 4). Interpretation of cord blood levels may thus be confounded by maternal adiposity and alteration in placental function. For example, diabetes and inflammation increase placental production of leptin and IL-6 (5, 6), two hormones with structural similarities (7), but very different effects on neurodevelopment (8, 9).

Leptin is a pleiotropic hormone that is a key regulator of energy balance through the hypothalamic-pituitary-adrenal axis. As an adipokine, leptin is found in higher levels in obese individuals, including obese pregnant women (10–13). While maternal leptin can be transferred across the placenta, fetal leptin levels only weakly correlate with maternal levels (14–16), consistent with the presence of redundant sources to provide leptin to the fetus, including the placenta itself (17).

Cord blood leptin has been associated with reduced insulin sensitivity of the fetus (15), and when adjusted for the confounding effects of maternal BMI and birth weight, increased cord blood leptin levels have been inversely related to childhood lean mass index and directly correlated with adiposity, but the associations have attenuated with age (14, 18). In animal models, excessive leptin exposure early in life can lead to a state of persistent leptin resistance and obesity in adulthood while leptin deficiency can interfere with organ growth and neurodevelopmental outcomes (19, 20). In many respects, the growth promoting and signaling effects of leptin mirror those of insulin (21).

Beyond its well characterized metabolic and trophic effects, leptin has an increasingly recognized role in inflammation (22, 23). From a structural standpoint, leptin is similar to acute phase reactants, including the proinflammatory cytokine interleukin-6 (IL-6) (7), and leptin levels are rapidly increased by multiple cytokines (24, 25). Elevation in IL-6 levels is often found in cord blood of infants affected by maternal chorioamnionitis (26–28), suggesting leptin could be similarly impacted. In contrast to the stimulatory effects of IL-6 on leptin, physiologic-range hyperleptinemia has been shown to attenuate the IL-6 response to endotoxemia, suggesting cord blood IL-6 levels could be attenuated in large for gestational age infants that are characteristically hyperleptinemic (29).

Beyond acute inflammatory cascades, IL-6 has recently gained attention as a potential biomarker of the chronic inflammation that contributes to the development of diabetes or obesity (30, 31), but the potential for maternal diabetes to impact perinatal development through exposure to IL-6 has not been defined. Concerning preclinical findings that include increased placental IL-6 production followed by the development of neuropathology in a maternal immune activation model and offspring behavioral alterations and increased adiposity following isolated maternal IL-6 administration alone emphasize the importance of assessing not just leptin but also IL-6 levels across a variety of perinatal conditions (8, 32, 33). Because the two adipokines can have both augmentative and counterbalancing effects on different types of inflammation, for example leptin induces Th1 responses while IL-6 inhibits them, there is a critical need to obtain correlative clinical data (34–36).

The goal of this study was to evaluate the differential impact of growth, maternal diabetes, and chorioamnionitis on umbilical cord blood leptin and IL-6 levels. We utilized a high risk pregnancy cohort enriched with perinatal morbidities of interest to test the hypothesis that there is a positive correlation between cord blood IL-6 and leptin with progressively increased levels in pregnancies complicated by maternal diabetes and chorioamnionitis.

## Materials and Methods

### Patient Selection

Using the University of Iowa IRB-approved Maternal-Fetal Tissue Bank database (IRB#200910784) (37), term infants were identified that met the inclusion criteria: birth following induction of labor and/or birth to a mother with diabetes during pregnancy. Plasma samples and corresponding clinical information were obtained for 104 infants (68 born following induction of labor and 36 born to women with diabetes). This study design was chosen to enrich the proportion of pre-specified morbidities, including large for gestational age birth weights, chorioamnionitis and maternal diabetes. Exclusion criteria included multiple gestation, fetal anomalies, maternal age less than 18 years, or inability to provide informed consent. The sample size was chosen to provide adequate power to detect moderate effect sizes (r=0.25) by simple linear regression and large effect sizes (f=0.4) by multiple regression models including up to 8 predictors of cord blood leptin (maternal obesity, maternal diabetes, maternal BMI, maternal weight gain, chorioamnionitis or sepsis, birth weight, cord blood insulin, and cord blood IL-6).

### Clinical Data and Sample Collection

Maternal and infant data were collected from the electronic medical record (Epic, Verona, WI, USA). Maternal data included age at delivery, first trimester weight (at first presentation to obstetric care), weight gain during pregnancy, maternal age, and first trimester body mass index (BMI), later categorized as normal (18.5-24.9), overweight (25.0-29.9), or obese (>30). Data were also collected on the presence of chorioamnionitis or diabetes, including gestational onset diabetes mellitus treated with diet or medication. Infant data included sex, birth weight, gestational age, and newborn antibiotic use. Umbilical cord blood samples were collected at the time of delivery into cord blood collection bags from Fenwal Scientific (Fenwal Inc., Lake Zurich, Illinois, USA). Plasma was separated, snap frozen, and stored at -80 degrees Celsius until further analysis.

### Plasma Analysis

Total protein content was quantified using the Pierce BCA protein assay (Pierce Biotechnology, Rockford, IL, USA). Leptin, insulin and IL-6 were quantified in duplicate using a custom multiplex panel (EMD Millipore, St Charles, MO, USA) on a BioRad analysis system (Bio-Rad Laboratories, Inc, Hercules, CA, USA) according to manufacturer’s instructions. The assay detects minimum concentrations of 41 pg/ml for leptin, 87 pg/ml for insulin, and 11 pg/ml for IL-6. Cord blood levels were normalized to total protein content to correct for dilution that occurs during the harvest of cord blood into the anticoagulated collection system.

### Statistical Analysis

All statistical analyses were performed with SigmaPlot 14.0 software (Systat Software, Inc, California) and confirmed with SPSS (Version 25.0, IBM Corp., Cary, NC). Data are expressed as mean +/- standard deviation. One-way analysis of variance and univariate linear regression were used to determine significant associations between hormone levels and categorical or continuous variables, respectively. A P value of <0.05 was considered statistically significant. When significant associations were present on univariate analysis, multiple regression was performed to identify independent predictors of hormone levels.

## Results

Demographic data and cord blood protein levels are summarized in **Table 1**. In this cohort, mean maternal BMI in the first trimester (30.4 kg/m^2^) exceeded the 30 kg/m^2^) cutoff typically used to define obesity. Mean maternal weight gain (12 kg) exceeded the recommendation for women with an initial BMI above 25 kg/m^2^), and 45% of women had excessive weight gain during pregnancy. Mean birth weight was 3524 grams, and when corrected for gestational age and sex, this represented the 57^th^ percentile.

**Table 1:** Patient characteristics and hormone levels in cord blood samples.

	Mean ± SD
Maternal age, years	31.6 ± 5.5
Maternal BMI, kg/m2	30.4 ± 9.8
Maternal weight gain, kg	12.2 ± 6.9
Gestational age, weeks	39.5 ± 1.1
Infant’s birth weight, grams	3524 ± 522
Total protein (TP), mg/ml	28 ± 10
Leptin, pg/ml	10499 ± 10486
Leptin/TP, pg/mg	436 ± 514
Insulin, pg/ml	391 ± 509
Insulin/TP, pg/mg	15 ± 19
IL-6, pg/ml	25 ± 45
IL-6/TP, pg/mg	0.91 ± 1.24

By linear regression, cord blood leptin significantly correlated with cord blood insulin (R=0.50, P<0.001). There was no correlation between IL-6 and either leptin (R=0.08, P=0.40) or insulin (R=-0.02, P=0.86). Maternal BMI significantly correlated with leptin (R=0.30, P<0.01) and insulin (R=0.44, P<0.001), but not IL-6 (R=-0.04, P=0.67). Maternal weight gain was not associated with leptin (R=0.07, P=0.45), insulin (R=0.04, P=0.67) or IL-6 (R=-0.07, P=0.47). Birth weight significantly correlated with leptin (R=0.37, P<0.001) and insulin (R=0.27, P<0.01), but not IL-6 (R=-0.18, P=0.07).

The prevalence of the perinatal outcomes of interest, and their associations with cord blood proteins are provided in **Table 2**. Maternal obesity, which was present in 39% of the cohort, was associated with a more than 2-fold increase in cord blood insulin. Likewise, maternal diabetes, which was present in 45% of the cohort, was associated with significant increases in cord blood leptin and insulin, but not IL-6. In contrast, clinically diagnosed maternal chorioamnionitis was associated with a 2-fold increase in IL-6. Antibiotics were prescribed for all 12 infants born to women diagnosed with chorioamnionitis and another 13 infants with suspected sepsis. The infants that received antibiotics had 2-fold increased IL-6 in their cord blood. There was no association between maternal chorioamnionitis or neonatal antibiotic use with cord blood leptin or insulin.

**Table 2:** Perinatal risk factors and their association with cord blood protein levels, reported as pg/mg total protein.

	N (%)	Leptin, pg/mg	Insulin, pg/mg	IL-6, pg/mg
Maternal BMI				
Normal	38 (37)	305 ± 230	10 ± 10	0.8 ± 0.9
Overweight	25 (24)	475 ± 450	12 ± 16	1.1 ± 1.5
Obese	41 (39)	535 ± 695	22 ± 24*	0.9 ± 1.3
Maternal Diabetes				
No	57 (55)	302 ± 289	7 ± 7	1.0 ± 1.2
Yes	47 (45)	599 ± 664*	26 ± 23*	0.7 ± 1.2
Chorioamnionitis				
No	92 (88)	436 ± 524	16 ± 20	0.8 ± 1.0
Yes	12 (12)	439 ± 452	11 ± 10	1.7 ± 2.4*
Newborn Antibiotics				
No	79 (76)	468 ± 557	15 ± 18	0.7 ± 0.6
Yes	25 (24)	338 ± 336	17 ± 22	1.4 ± 2.2*

*P<0.05.

Of the pre-specified risk factors, only maternal chorioamnionitis and neonatal antibiotic use were associated with IL-6 levels. In contrast, multiple factors were associated with leptin and insulin, including maternal diabetes, maternal BMI and infant’s birth weight. By multivariate regression (**Table 3**), birth weight was the strongest predictor of cord blood leptin (P<0.001), while maternal diabetes and BMI were the strongest predictors of cord blood insulin (both P<0.001).

**Table 3:** Generalized linear model with leptin or insulin as the dependent variable, maternal diabetes as a factor and maternal BMI or birth weight as potential covariates.

	Leptin		Insulin	
	Wald Chi-Squared	P	Wald Chi-Squared	P
Maternal Diabetes	5.1	0.02	27	<0.001
Maternal BMI	4.7	0.03	14.5	<0.001
Birth weight	12.5	<0.001	4.4	0.04

**Figure 1:**
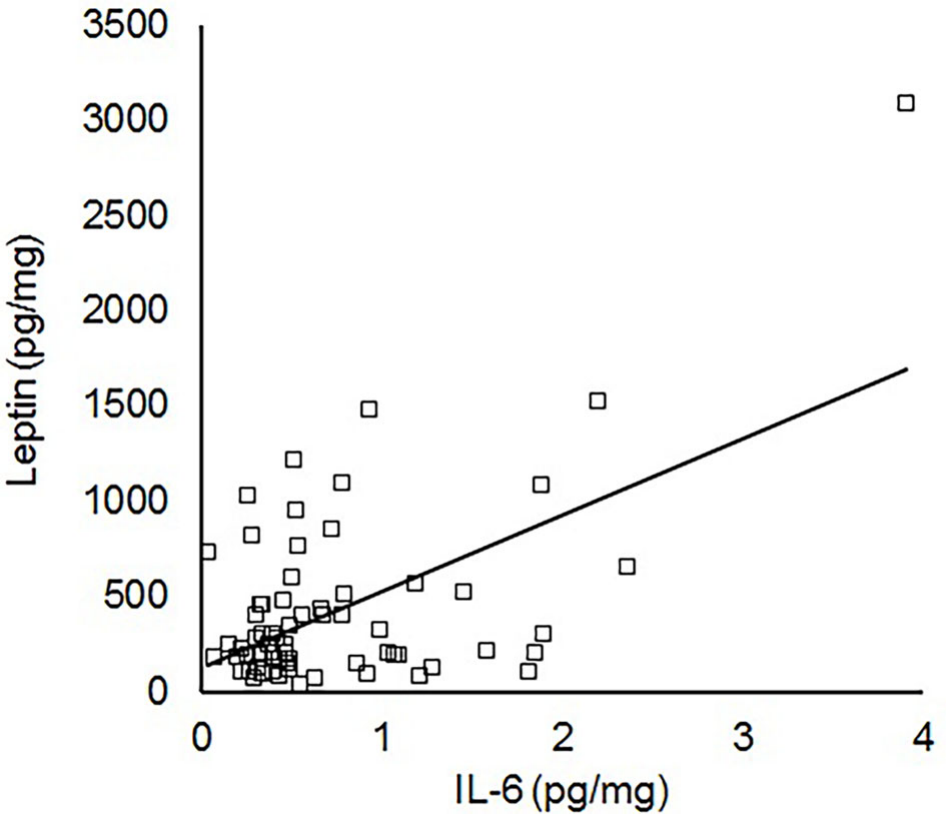
Linear correlation between IL-6 and leptin within the cord blood of appropriate for gestational age term infants without sepsis (N=67, R=0.56, P<0.001).

To further assess IL-6 levels in the absence of the confounding effects of acute perinatal infection, additional analyses were performed after excluding the 25 infants that received antibiotics for suspected sepsis. By linear regression, cord blood IL-6 in those without suspected infection was significantly correlated with cord blood leptin (N=79, R=0.39, P<0.001) but not insulin (R=0.10, P=0.37). There was still no association between IL-6 and birth weight (R=0.01, P=0.96), but the association between leptin and IL-6 was strengthened when the analysis was restricted to appropriate for gestational age infants without sepsis (N=67, R=0.56, P<0.001, **Figure 1**) or infants without sepsis with birth weights at the 25^th^ to 75^th^ percentile (N=34, R=0.71, P<0.001). In the absence of sepsis, there was no association between IL-6 and maternal diabetes (P=0.49) or obesity (P=0.77).

## Discussion

The purpose of this study was to evaluate the association of two risk factors for impaired neonatal development, maternal diabetes and chorioamnionitis, with umbilical cord blood levels of two hormones that may contribute to long-term neurodevelopmental outcomes, leptin and IL-6. The results demonstrate that birth weight is the strongest predictor of cord blood leptin, with maternal BMI and diabetes having a greater influence on insulin than leptin. Cord blood leptin was unaltered in the presence of clinically diagnosed infection but correlated with IL-6 in the absence of infection. In contrast, IL-6 was predominately associated with maternal chorioamnionitis, rather than birth weight or diabetes. This is the first study we are aware of that demonstrates an association between cord blood IL-6 and leptin in appropriate for gestational age infants without sepsis.

Birth weight remains the most widely available predictor of the long-term complications of potentially adverse prenatal exposures (38). In the presence of maternal obesity and diabetes, increased birth weight could be a consequence of the trophic effects of hormones, including insulin and leptin, as well as a manifestation of either genetic predisposition or enhanced nutrient availability. As expected, we noted the highest insulin levels in infants of diabetic mothers. This is typically ascribed to increased insulin production by the fetal pancreas in response to maternal hyperglycemia, although transplacental insulin passage may contribute (1, 39). As noted by others, we observed an independent association between maternal BMI and cord blood insulin which may reflect a role for subclinical insulin resistance in overweight or obese individuals that have not been diagnosed with diabetes (39–41).

As an adipokine that is also produced by the placenta, leptin’s relationship with maternal and newborn anthropometrics is inherently more complex than that of insulin. Although we did not directly assess adiposity or placental morphology, birth weight and maternal BMI were both associated with cord blood leptin levels. Because our study was focused on term infants that would be expected to have expanding adipose depots, endogenous leptin production would be expected to significantly contribute to circulating leptin levels (42), and on multivariate analysis, birth weight was the strongest predictor of cord blood leptin. Our results are consist with the observations of Gross and colleagues as well as Persson and colleagues that demonstrated higher cord blood leptin in infants of diabetic mothers with a strong association with birth weight; those investigations also each replicated the results of others in showing no association between maternal leptin and cord blood leptin, again suggesting an important role for birth weight-related alterations in leptin production or metabolism (43–45). The correlation we observed between cord blood insulin and leptin is consistent with known role of insulin in the regulation of adipocyte leptin production and the previous reports of others (46, 47).

We compared leptin and IL-6 level across two common perinatal conditions that are coupled with inflammation, diabetes and chorioamnionitis. The association of leptin with diabetes but not chorioamnionitis is consistent with a more robust role for leptin in endocrine than infectious conditions, and a relatively diminished role for leptin in perinatal inflammatory cascades. That contrasts with the significant elevation in IL-6 that we found associated with chorioamnionitis or neonatal sepsis but not diabetes; this finding of increased IL-6 with presumed infection increases confidence that if leptin played a significant augmentative role in perinatal infection-related inflammation within our cohort, we would have noted altered cord blood levels or a correlation between IL-6 and leptin. Like leptin, IL-6 is an adipokine, but in contrast to leptin, IL-6 production by adipocytes typically requires an active inflammatory response that may be blunted at later stages of pregnancy (48, 49). Gestational diabetes-related immune dysregulation has been shown to lead to increased maternal IL-6 with enhanced placental leptin production but a paradoxical reduction in the IL-6 levels of the LGA offspring (30), possibly related to the hyperleptinemia that is typically seen in LGA infants (29).

This is the first investigation we are aware of that assessed the levels of leptin and IL-6, two adipokines with overlapping signaling pathways but divergent implications for neurodevelopment, in the context of common pregnancy complications. Leptin, a neurotrophic hormone, was increased primarily in association with birth weight and secondarily by maternal obesity and diabetes. IL-6, a potential neurotoxin, was increased in the presence of presumed perinatal infection but not maternal obesity or diabetes. These results clarify the unique predispositions towards adult disease that can follow major maternal morbidities and suggest leptin may not be biologically positioned to counterbalance the effects of IL-6 in the presence of chorioamnionitis. It is possible that our use of a high-risk cohort with a high rate of maternal diabetes and obesity may limit generalization to cohorts with a lower incidence of maternal morbidities. Future studies are also needed to assess the long-term implications of these and other hormones and to assess them at earlier gestational ages, when relatively lower fetal leptin production may unmask potential maternal obesity or diabetes-related alterations in IL-6 exposure during critical neurodevelopmental windows.

## Data Availability Statement

The raw data supporting the conclusions of this article will be made available by the authors, without undue reservation.

## Ethics Statement

The studies involving human participants were reviewed and approved by the University of Iowa’s Institutional Review Board. The patients/participants provided their written informed consent to participate in this study

## Author Contributions

LV, BS, DS, MS, DB, DD, and RR contributed to conception and design of the study. LV, BS, DS, and DB organized the database. LV, BS, and RR performed the statistical analysis. LV and BS wrote the first draft of the manuscript. DS and RR wrote sections of the manuscript. All authors contributed to manuscript revision, read, and approved the submitted version.

## Conflict of Interest

The authors declare that the research was conducted in the absence of any commercial or financial relationships that could be construed as a potential conflict of interest.

## Publisher’s Note

All claims expressed in this article are solely those of the authors and do not necessarily represent those of their affiliated organizations, or those of the publisher, the editors and the reviewers. Any product that may be evaluated in this article, or claim that may be made by its manufacturer, is not guaranteed or endorsed by the publisher.
